# New Sustainable Synthetic Routes to Cyclic Oxyterpenes Using the Ecocatalyst Toolbox

**DOI:** 10.3390/molecules26237194

**Published:** 2021-11-27

**Authors:** Camille Bihanic, Arthur Lasbleiz, Morgan Regnier, Eddy Petit, Pierre Le Blainvaux, Claude Grison

**Affiliations:** 1 Laboratory of Bio-inspirated Chemistry and Ecological Innovations (ChimEco), UMR 5021, CNRS—University of Montpellier Cap Delta, 1682 rue de la Valsière, 34790 Grabels, France; camille.bihanic@umontpellier.fr (C.B.); arthur.lasbleiz@ens-lyon.fr (A.L.); morgan.regnier@enscm.fr (M.R.); 2 European Institute of Membrane (IEM), UMR 5635—University of Montpellier 163 rue Auguste Broussonet, 34090 Montpellier, France; eddy.petit@umontpellier.fr; 3 BioInspir Cap Delta, 1682 rue de la Valsière, 34790 Grabels, France; pierre.leblainvaux@bioinspir.com

**Keywords:** ecocatalysis, green chemistry, oxyterpenes, ß-pinene, water lettuce

## Abstract

Cyclic oxyterpenes are natural products that are mostly used as fragrances, flavours and drugs by the cosmetic, food and pharmaceutical industries. However, only a few cyclic oxyterpenes are accessible via chemical syntheses, which are far from being ecofriendly. We report here the synthesis of six cyclic oxyterpenes derived from ß-pinene while respecting the principles of green and sustainable chemistry. Only natural or biosourced catalysts were used in mild conditions that were optimised for each synthesis. A new generation of ecocatalysts, derived from Mn-rich water lettuce, was prepared via green processes, characterised by MP-AES, XRPD and TEM analyses, and tested in catalysis. The epoxidation of ß-pinene led to the platform molecule, ß-pinene oxide, with a good yield, illustrating the efficacy of the new generation of ecocatalysts. The opening ß-pinene oxide was investigated in green conditions and led to new and regioselective syntheses of myrtenol, 7-hydroxy-α-terpineol and perillyl alcohol. Successive oxidations of perillyl alcohol could be performed using no hazardous oxidant and were controlled using the new generation of ecocatalysts generating perillaldehyde and cuminaldehyde.

## 1. Introduction

Oxygenated terpenoids, or oxyterpenes, are secondary metabolites of plant origin with a wide range of chemical structures. Their strong fragrances, flavours and pharmacological effects for some of them, make them attractive yet challenging targets for total synthesis by cosmetic, food and pharmaceutical industries.

Indeed, a few cyclic oxyterpenes derived from ß-pinene oxide exhibit interesting properties. For example, myrtenol shows anti-inflammatory [1] and antianxiolytic activities [2]. It can also be used against myocardial ischemia-reperfusion lesions [3].

Perillyl alcohol, which can be found in essential oil of lavender and peppermint, shows anticancer [4,5], analgesic [6], antibacterial and antifungal properties [7]. While it could be also used as a drug against Alzheimer’s Disease [8] and malaria [9], it is commonly used in the fragrance industry [10,11].

Perillaldehyde is largely found in the essential oil of an aromatic plant, *Perilla frutescens*, also known by its Japanese name shiso. The essential oil of shiso, and the molecule of perillaldehyde itself, are widely used in the fragrance and food-industries [12]. Perillaldehyde also exhibits anti-inflammatory [13], neuroprotective [14], antidepressant [15], antifungal [16] and antibacterial [17] properties.

Cuminaldehyde is mostly found in the plant species *Cuminum Linn* [18,19,20] and gives the characteristic flavour of cumin to its essential oil, as its mass fraction is 20–40% [21]. In addition to the wide use of the essential oil of cumin and cuminaldehyde in cosmetics and perfumery, cuminaldehyde exhibits antidiabetic, anticancer, neuroprotective, anti-inflammatory, antimicrobial and antifungal properties [22,23].

Although these cyclic oxyterpenes have various applications, only a few of them are accessible via chemical syntheses, and require the use of toxic or hazardous reagents, stoichiometric amounts of oxidants or environmentally unsustainable conditions. We present here the synthesis of five cyclic oxyterpenes: myrtenol, 7-hydroxy-α-terpineol, perillyl alcohol, perillyladehyde and cuminaldehyde, via the platform molecule, ß-pinene oxide, derived from the epoxidation of ß-pinene.

The selective epoxidation of ß-pinene is challenging because of the tensions of the pinane ring system and the exo position of the double bond on the one hand, and the high reactivity of the corresponding epoxide on the other hand [24]. Thus, the oxidation of β-pinene can lead to several oxidation products, including epoxy, [25] which can itself rearrange into several by-products [26,27,28]. Indeed, β-pinene oxide is very reactive due to the many steric constraints imposed by the three rings. It is, therefore, very sensitive to acidic and basic media and is easily rearranged by opening the pinane ring and/or the epoxy ring. The rearrangement of the epoxide gives rise to several products, mainly myrtanal and myrtenol, by opening the epoxide ring, and perillyl alcohol and 7-hydroxy-α-terpineol by opening the bicyclic structure [29]. Due to its fragility, the preparation and the selective opening of β-pinene oxide to one of these compounds present a real challenge.

The preparation of this library of cyclic oxyterpenes based on the platform molecule, ß-pinene oxide, relies on only using green and sustainable chemistry.

The Grison group has shown that remediation phytotechnologies, such as phytoextraction [30,31,32], rhizofiltration [33,34,35] and biosorption [35,36,37,38], generate biomass rich in transition metals that can be turned into innovative catalysts, called ecocatalysts [39,40,41]. Using the concept of ecocatalysis, we advanced the aim that only natural or biosourced catalysts would be used for preparing the cyclic oxyterpenes.

Leaves of *Grevillea gillivrayi*, an endemic tree from New Caledonia, have been used for preparing ecocatalysts and proved their efficacy in several syntheses [42]. However, considering the location and natural abundance of leaves of *G. gillivrayi*, other sources of Mn-rich biomass were sought. Roots of water lettuce, *Pistia stratiotes*, were found to be an excellent alternative source of Mn-rich biomass [43], as the aquatic plant is an invasive alien species (IAS), hence abundant and easy to harvest. At the same time, the harvest of *Pistia stratiotes* could contribute to eradication, or controlling the species abundance.

A new generation of ecocatalysts derived from Mn-rich water lettuce was hence considered here and compared to the previous ecocatalysts of *G. gillivrayi*. The new ecocatalysts were prepared via green processes and characterised by MP-AES and XRD analyses. They were tested through the epoxidation of ß-pinene into ß-pinene oxide. The selective opening of ß-pinene oxide was investigated in mild conditions for the synthesis of myrtenol, 7-hydroxy-α-terpineol and perillyl alcohol. Finally, successive oxidations of perillyl alcohol were tested using an innocuous and eco-friendly oxidant, and the new generation of ecocatalysts for the synthesis of perillaldehyde and cuminaldehyde.

## 2. Results and Discussion

### 2.1. The Ecocatalyts Toolbox

#### 2.1.1. Preparation of the Ecocatalysts

**Figure 1:**
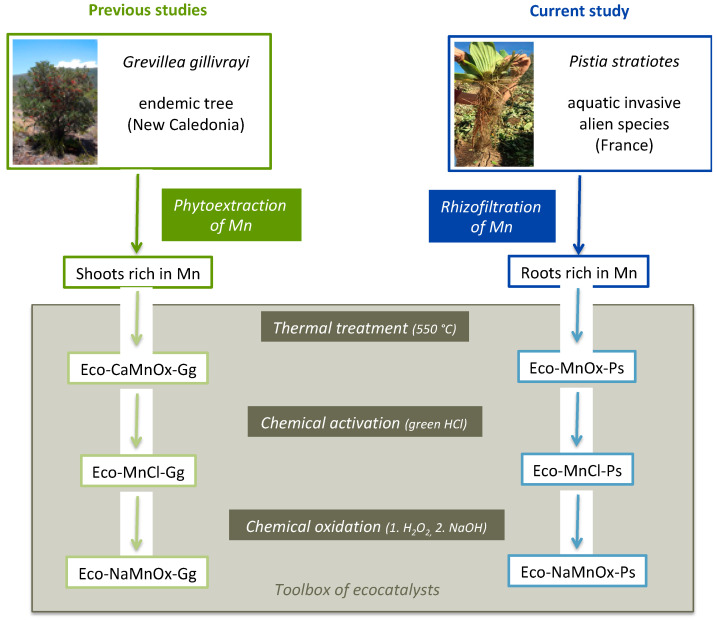
Preparation of ecocatalysts bearing different chemical properties.

Considering the diversity of reactions leading to oxyterpenes from ß-pinene, several classes of ecocatalysts bearing different chemical properties, and hence different reactivities, were considered for preparation (Figure 1).

Roots from *Pistia stratoties*, an aquatic invasive alien species (IAS) in France, were chosen for providing the biomass to transform into ecocatalysts. *P. stratoties* showed the spontaneous ability to concentrate heavy metals, especially Mn, in roots by rhizofiltration. Indeed, roots of *P. stratoties* in the Rhône canal in France presented high concentrations of Mn (Table 1, entries 1 & 5).

As has been done previously with G. gillivrayi [31], the Mn-rich biomasses of *P. stratiotes* could be transformed into three classes of ecocatalysts (Figure 1). A first class of ecocatalysts was obtained by grinding the air-dried leaves or roots into a 1.5 mm powder, which was then heated to 550 °C, leading to formation of Eco-MnOx-Ps. This first class of ecocatalysts was transformed using green hydrochloric acid [44] into Eco-MnCl-Ps. This second class of ecocatalysts was further oxidised using hydrogen peroxide followed by an alkaline treatment, producing Eco-NaMnOx-Ps.

#### 2.1.2. Characterization of the Ecocatalysts

The elemental composition of the ecocatalysts was determined by MP-AES analyses (Table 1). As expected, and due to the Mn-rich biomass, Mn was the most or second most abundant element in the ecocatalyst. Moreover, other physiological elements as Ca, Mg and K were found in significant amounts, since the biomass was derived from an aquatic plant.

X-Ray Powder Diffraction Analyses were then performed to characterize the complexes found in the ecocatalysts (Table 2). The complexes involving Mn were different among the classes of ecocatalysts. Eco-MnOx-Ps, which was obtained after a simple thermal treatment of the biomass, shows two complexes of Mn: Mn(II) oxide and a mixed complex of Mn(III) and Mn(IV) suggesting oxidative properties in catalysis (Table 2, entry 1). Eco-MnCl-Ps, obtained after a hydrochloride treatment, shows a complex of mixed potassium/sodium Mn (II) chloride (Table 2, entry 2). Interestingly, this salt, which cannot be obtained by a chemical synthesis, has a similar hardness to MnCl_2_ in HSAB theory, while having a milder Lewis acidity [45].

Moreover, a comparison between the ecocatalysts derived from *P. stratoties* and *G. gillivrayi* shows different complexes of Mn, suggesting that the plant species leaves a vegetal footprint, specific of the species, in the ecocatalyst (Table 2, entries 3 & 4).

TEM images of Eco-MnOx-Ps, Eco-NaMnOx-Ps and Eco-MnCl-Ps exhibited different particles with various shapes (see Supplementary Materials). Small round particles of about 3–5 nm of diameter with Eco-NaMnOx-Ps, lamellar particles of about 2–5 nm width over 10–30 nm length with Eco-MnOx-Ps and Eco-MnCl-Ps, respectively, as well as thin layer-shape particles, were blended together into a matrix.

### 2.2. Syntheses of Oxyterpenes

**Figure 2:**
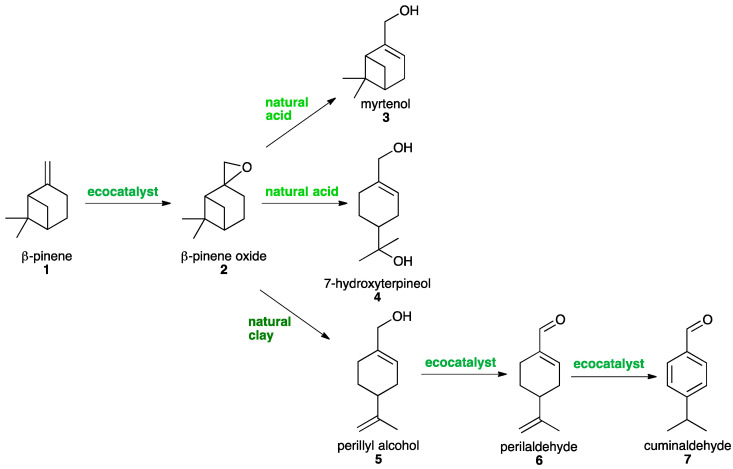
Green synthesis of oxyterpenes using ecocatalysis.

The green and sustainable synthesis of five oxyterpenes **3**–**7** of diverse industrial interests was considered through the epoxidation of ß-pinene **1** into the platform molecule **2**, ß-pinene oxide, and its selective opening (Figure 2).

#### Selective Epoxidation of ß-Pinene **1** into ß-Pinene Oxide **2**

The epoxidation of ß-pinene **1** into ß-pinene oxide **2** is far from trivial since this reaction can lead to nine major products [46,47]. Moreover, the low stability of ß-pinene oxide **2**, which can be degraded into about 10 side-products [48,49], is also a key parameter for choosing the reaction conditions. Indeed, classic conditions of epoxidation require organic peroxides or percarboxylic acids such as *m*-CPBA [50], which lead to a significant degradation rate of ß-pinene oxide **2** and/or are questionable in terms of safety considerations and waste production. Ideally, eco-friendly oxidants such as hydrogen peroxide in combination with Mn constitute effective catalysts for epoxydation. For the epoxidation of β-pinene, different Mn catalytic systems based on the joint use of H_2_O_2_ and NaHCO_3_ have been described in the literature. A yield of 76% can be obtained with manganese sulfate [51] and 84% with Mn (II) dispersed on graphene oxide [52]. However, in both cases, a toxic solvent, DMF, is used. Sodium perborate was also used, but wastes containing boron are now restricted by REACH regulations [53]. Catalysts based on expensive, scarce and/or toxic metallic elements, such as palladium, gold, niobium or tungsten have been described but resulted in either low conversion or low selectivity for epoxide, as they led to competitive oxidation of the allylic position [26,46,47].

The right balance between the selectivity of epoxidation and the stability of the product formed, while respecting an environmentally friendly process, was hence considered. The previous generation of ecocatalyst derived from *G. gillivrayi*, and presenting mild oxidative properties, Eco-CaMnOx-Gg, was first tested as a biosourced catalyst with a mild base (sodium bicarbonate,) and a green co-oxidant (hydrogen peroxide) in a mixture of green solvents (acetone and water) (Table 3, entry 1). Despite high conversion, the yield did not exceed 33%. The new generation of ecocatalyst derived from *P. stratiotes*, and also presenting mild oxidative properties, Eco-MnOx-Ps, was tested in the same conditions and resulted in total conversion and the best yield of 63% (Table 3, entry 2). Increase in the catalytic loading and the use of a Lewis acid support, MK10, were tested but led to more degradation of ß-pinene oxide **2** (Table 3, entries 3 & 4). Another class of ecocatalyst derived from *P. stratiotes* and presenting Lewis acid properties instead of oxidative properties, Eco-MnCl-Ps, was also tested (Table 3, entry 5) but did not improve the yield and also produced 63% of ß-pinene oxide **2**. The use of MK10-supported Eco-MnCl-Ps was also tested but decreased the yield due to product degradation (Table 3 entry 6).

**Figure 3:**
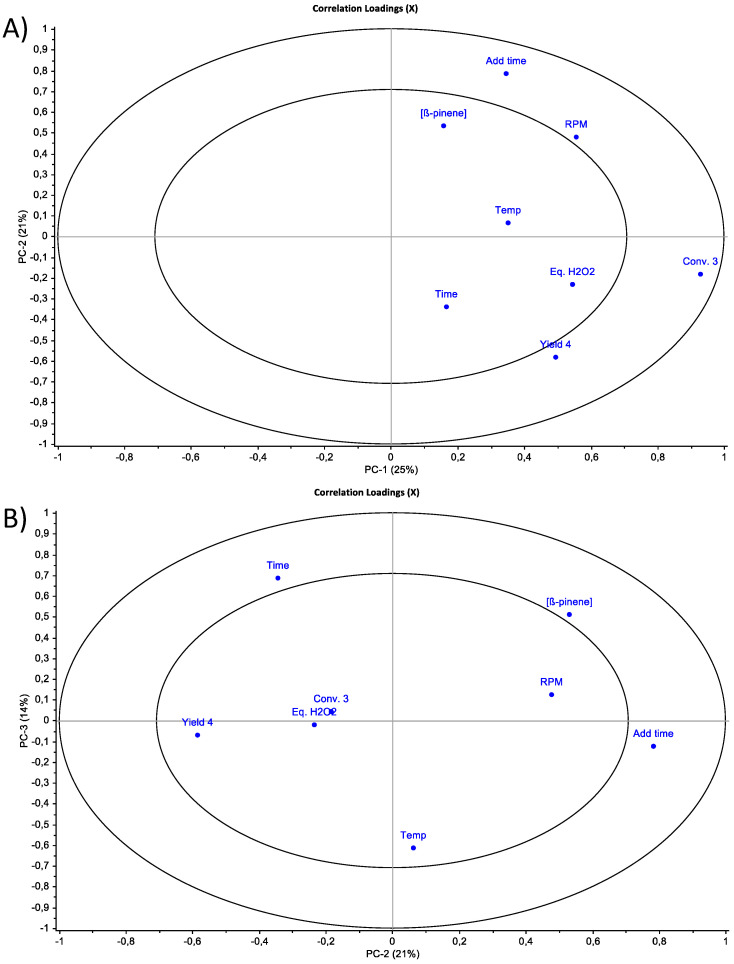
Principal Component Analyses of the experimental variables tested for optimising the epoxidation of ß-pinene **1** into ß-pinene oxide **2**. (**A**) All tested variables were used. (**B**) Refined PCA using key variables determined in the first PCA.

Considering the moderate yield obtained here, other experimental parameters were investigated and then analysed using Principal Component Analyses (PCA). The temperature, reaction time, stirring speed, quantity of ß-pinene **1**, quantity of peroxide hydrogen, and time of addition of peroxide hydrogen were tested using Eco-MnOx-Ps as the catalyst. A first PCA was modelled using all these parameters together (Figure 3A). It shows that the stirring speed and quantity of peroxide hydrogen had an impact on the conversion and yield of epoxidation. A second PCA was refined using these two parameters (Figure 3B). This shows that the time of addition of hydrogen peroxide and the concentration of ß-pinene **1** are anti-correlated to the yield. Hence hydrogen peroxide was added drop-wise to the reaction mixture and the concentration of ß-pinene **1** was low.

Despite this accurate study of the reaction parameters, it was not possible to improve the yield and selectivity towards the formation of ß-pinene oxide **2**.

Although the yield of this reaction remained moderate, it was better than gold, palladium, molybdenum, niobium, titanium and tungsten systems, and equivalent to the yield using other manganese catalysts [47,54,55,56,57]. However, the conditions described here are eco-friendlier. Indeed, the ecocatalyst (Eco-MnOx-Ps) and solvents (acetone/H_2_O) can be derived from easily available renewable feedstock. The low loading of Mn (0.005 eq.), the absence of ligand, the moderate time (2 h) and temperature (30 °C) illustrate the performances of the catalytic system as a greener and sustainable alternative to classic catalysts.

### 2.3. Selective Opening of ß-Pinene Oxide ***2***

The opening of the platform molecule **2**, ß-pinene oxide, can lead to about 10 products according to the conditions of reaction [46,47]. Considering the mechanism of opening, myrtenol **3**, the constrained bicyclic compound would be the kinetic product. Perillyl alcohol **5**, the six-membered ring compound, should be lower in energy and thermodynamically more stable. However, Corma et al. have shown that myrtenol is not an intermediate that is rearranged further into perillyl alcohol, but the two products come from different pathways. The opening of ß-pinene oxide **2** can lead to perillyl alcohol **5**, based on the rearrangement of the bicyclic carbon atom skeleton, followed by epoxide ring opening. The opening of ß-pinene oxide **2** can lead to myrtenol **3** following a b-elimination mechanism [24].

Specific conditions were tested for selectively opening the epoxide into myrtenol **3**, 7-hydroxy-α-terpineol **4** and perillyl alcohol **5**.

#### 2.3.1. Synthesis of Myrtenol **3**

The synthesis of myrtenol **3** based on the opening of ß-pinene oxide **2** has not been extensively reported in the literature. Indeed, myrtenol **3** is mostly considered a side-product of the formation of perillyl alcohol **5** by the action of a Brønsted acid on ß-pinene oxide **2** [24,25,26,27]. Several Brønsted acid catalysts were tested to favour the formation of myrtenol **3** instead of perillyl alcohol **5** (Table 4). Among the biosourced catalysts tested here, betaine hydrochloride, which has the strongest acidity, gave the best selectivity towards myrtenol **3** (Table 4 entry 1).

These results are surprising given the studies of Corma et al. [24], which suggested the use of weak acids. Betaine hydrochloride can be advantageously used to replace other Brønsted acids in terms of selectivity and sustainability [24,25,26].

#### 2.3.2. Synthesis of 7-Hydroxy-α-terpineol **4**

To our knowledge, the synthesis of 7-hydroxy-α-terpineol **4** has never been reported in the literature as a targeted product but has been observed as a side-product during the formation of perillyl alcohol **5** in the presence of water, with a maximum yield of 25% [50]. Here is reported, for the first time, the synthesis of 7-hydroxy-α-terpineol **4**, which can be reached in a one-pot synthesis from ß-pinene **1,** or sequentially from ß-pinene oxide **2** using Brønsted acids in a mixture of water and acetone (Table 5).

The optimization was first carried out using HCl (Table 5 entries 1 & 2) and then tested with green biosourced acids (Table 5 entries 3–7). Three equivalents of oxalic acid, formic acid, betaine hydrochloride or thiamine hydrochloride led to similar high yields of 7-hydroxy-α-terpineol **4** in only one hour at moderate temperature and in green solvents.

#### 2.3.3. Synthesis of Perillyl Alcohol **5**

In the literature, perillyl alcohol **5** can be obtained with a yield of 47% using natural zeolite over several hours at 70 °C [28]. Another clay, hectorite, could improve the yield to 64% but using pyridinium nitrate in the presence of nitric acid [27]. Other catalytic systems, based on metal enriched zeolites or mesoporous materials, led to higher selectivities of 60–70%. However, these syntheses relied on the use of hazardous and toxic solvents [25,26].

**Figure 4:**
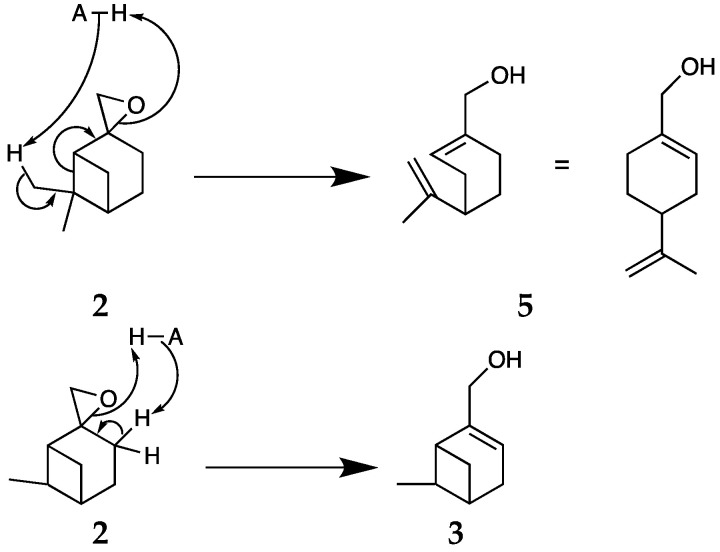
Mechanism for the opening of ß-pinene oxide into perillyl alcohol 5 and myrtenol 3 [24,25,26,27].

Considering the mechanism described by Corma et al. [24] (Figure 4), the formation of perillyl alcohol **5** is favoured by adjusting a combination of acid-base properties of the reaction medium.

Eco-MnCl-Ps, which presents both Lewis and Brønsted acid properties, was first tested in a basic and green solvent, CPME, but mainly led to the opening of epoxide prior to the rearrangement of the bicyclic carbon atom skeleton (Table 6, entry 1). Another green catalyst bearing both but weaker acid properties, the natural clay, Montmorillonite K10 (MK10), was used instead, and led to a better selectivity towards perillyl alcohol **5** (Table 6, entries 2–4). However, dimerization side-products were still observed in high quantity. Dilution of the catalyst and different catalytic loadings were tested (Table 6, entries 5–8). The highest dilution and moderate catalytic loading led to the best yield (Table 6, entry 6). Several tests on temperature and reaction time were performed but no further improvement was obtained.

Although the yield of this reaction was rather low, it was equivalent to the yields described in the literature and the conditions described here are eco-friendlier. Other described chemical procedures are dependent on the use of high temperatures (hydrothermal synthesis of zeolites), toxic solvents (N,N-dimethyl formamide, N-methyl pyrrolidone, N,N-dimethyl acetamide, dimethyl sulfoxide, dioxane [25,26] or hazardous reagents (nitric acid, [26,27], tin, [25], pyridinium nitrate [27]. Conversely, this work presents a method where none of these environmentally “unfriendly” parameters are needed, and which can, therefore, be considered “green” and cleaner that other reported processes.

### 2.4. Synthesis of Perillaldehyde ***6***

The oxidation of perillyl alcohol **5** into perillaldehyde **6** is not well documented in the literature. Li et al. reported the synthesis of perillaldehyde via the selective oxidation of perillyl alcohol by supported CrO_3_/SiO_2_ oxidant in CH_2_Cl_2_. However, CrO_3_ and CH_2_Cl_2_ are both toxic molecules regulated by REACH [58].

This transformation was studied using dioxygene in cyclohexane to allow its maximum dissolution. Previous studies on the oxidation of the allyl alcohol of geraniol into the aldehyde of geranial showed that the use of ecocatalyst derived from *G. gillivrayi*, Eco-NaMnOx-Gg, led to a quantitative yield [59]. Herein, Eco-CaMnOx-Gg and Eco-MnOx-Ps were tested for catalysing the synthesis of perillaldehyde **6** but did not give the desired product. An ecocatalyst with stronger oxidative properties was chosen instead, Eco-NaMnOx-Ps (Table 7, entries 2–8). After increase of catalytic loading, the use of four equivalents of a co-oxidant, CuO, was tested and gave the best yield of 66% (Table 7, entries 4). The co-oxidant alone was tested as a negative control and no reaction occurred (Table 7, entry 6). Since the conversion was not complete in 2 h, longer reaction times were tested but did not increase the yield and led to more degradation (Table 7, entries 7 & 8).

### 2.5. Synthesis of Cuminaldehyde ***7***

To our knowledge, we report the synthesis of cuminaldehyde **7** from perillaldehyde **6** for the first time. This transformation relies on the migration of the exocyclic double bond into the 6-membered ring, giving the intermediate *p*-menta-1,3-dien-7-al, which facilitates a final aromatisation. Considering this mechanism, an ecocatalyst bearing both Bronsted acid and oxidative properties, Eco-MnCl-Ps, was first chosen in solvent-free conditions (Table 8 entry 1). However, almost no desired product was formed.

A similar oxidation, the oxidation of citronellal into *p*-cymene, has been described using clays as catalysts [59]. MK10 and Eco-MnCl-Ps supported on MK10 were tested but no improvement was observed (Table 8, entries 2 & 3). Moreover, the intermediate, *p*-menta-1,3-dien-7-al, was never observed in these conditions, suggesting that the Brønsted acidity of MK10 is too weak and prevents the first step occurring. A stronger Brønsted acid, citric acid functionalised coffee grounds (coffee ground-CA), was added to Eco-MnCl-Ps and gave a better yield (Table 8, entry 5). As a control, coffee ground-CA was tested alone and led to no conversion (Table 8, entry 4).

Since Eco-MnCl-Ps used in the presence of coffee ground-CA led to main side-products derived from polymerisation of perillaldehyde **6**, dilution of the reagents was tested in a green solvent, CPME, and the highest dilution rate gave the best yield of 55% (Table 8, entry 9).

## 3. Materials and Methods

### 3.1. General Information

GC-MS analyses were performed on a Thermo Scientific™ Trace 1300 GC coupled with an ISQ QD quadrupole. The detection system was connected with a Thermo TG-5SILMS column (0.18 µm × 0.18 µm × 20 m). GC-FID analyses were performed on a similar column connected to a flame ionization detector (FID). In each case, hydrogen was used as carrier gas (1 mL.min^−1^), using the following temperature program: 80 °C isothermal (1 mn), 80 °C to 260 °C gradient at 40 °C.min^−1^, then 260 °C isothermal (1 mn).

The samples were prepared in ethyl acetate, and biphenyl was used as internal standard for GC-FID quantifications. Mass spectra were recorded in impact electronic mode at 70 V and identification was made by the NIST 14 database.

NMR spectra were recorded on a Brücker Avance III HD–400 MHz at room temperature. The ^1^H frequency was at 400 MHz and the ^13^C frequency was at 100 MHz. NMR quantifications were established by ^1^H quantitative analyses using an internal standard with a structure similar to the target-molecule.

Transmission Electron Microscopy (TEM) analyses were performed at 200 kV on a JEOL 2200 FS equipped with a CCD Gatan Ultrascan 4000 CCD (4092 × 4092 px2) at the MEA platform (University of Montpellier, Montpellier, France).

Product purification was made by Puriflash InterChim 430 coupled with a UV detector. A silica gel column was used with a mixture of cyclohexane:ethyl acetate as eluent at a flow rate of 30 mL/min.

Mineral composition of the catalysts was determined using an Agilent Technologies™ 4200 Microwave Plasma-Atomic Emission Spectrometer (MP-AES) coupled with a SPS4 autosampler. A One-Neb nebulizer was used. The samples (between 5 and 10 mg for solids, 2 mL for liquids) were digested in 6 mL of reversed aqua regia (1:2 hydrochloric acid (37%): nitric acid (65%)) under an Anton Paar Multiwave Go™ microwave-assisted digestion with the following program: 20 °C to 164 °C in 20 min then 10 min isothermal at 164 °C. Samples were filtered and then diluted to 0.2 g.L^−1^ in nitric acid (1%). Three analyses were carried out for each sample, multi element standards (calibration range between 0.1 and 10 ppm) and digestion blanks, in order to determine the standard deviation of the measurement.

X-Ray Powder Diffraction (XRPD) data measurements on samples dried at 100 °C for 2 h were conducted using a BRUCKER diffractometer (D8 Advance, with Cu K𝛼 radiation at 1.54086 Å) equipped with a Lynxeyes detector. Analyses were carried out at Institut Jean Lamour (University of Lorraine, Nancy, France).

### 3.2. General Procedures

#### 3.2.1. Preparation of the Ecocatalysts

##### Harvest of Pistia Stratiotes

Plants of *Pistia stratiotes* grow spontaneously in the Canal of Rhône River. Whole plants were harvested at Comps in September 2020. Roots were separated from the rest of the plant on site before being dried.

##### Transformation of Biomass into Eco-CaMnOx-Gg and Eco-MnOx-Ps

In a typical procedure, the leaves of *Grevillea gillivrayi* (75 g) or the roots of *Pistia stratiotes* (7.85 g) were air-dried at room temperature then ground into a powder with a granulometry of 1.5 mm. The powder was heated at 550 °C for 4 h, producing Eco-CaMnOx-Gg (3.75 g) and Eco-MnOx-Ps (2.64 g). On average, weight loss was 95% for *Grevillea gillivrayi* and 65% for *Pistia stratiotes*.

##### Preparation of Green HCl (6 M)

Leaves of sorrel or *Rumex acetosa* (38 g) were ground, slightly heated and stirred. An aqueous solution of oxalic acid was obtained (pH ≈ 2.5). The biomass was removed by filtration. Sodium chloride (10 g) was added to the mixture. The mixture was distilled using water as an azeotrope and produced 10 mL of a 6 M hydrochloric acid solution [44].

##### Preparation of Eco-MnCl-Gg and Eco-MnCl-Ps

In a typical procedure, Eco-CaMnOx (50 g) was introduced into a flask and green HCl (6 M; 500 mL) was added cautiously. The resulting suspension was heated up at 85 °C and stirred for 5 h. The suspension was then cooled down, filtered and washed three times with green HCl (6 M). Water was removed under reduced pressure and the obtained solid was dried at 85 °C for 24 h, producing Eco-MnCl, which was kept in a desiccator.

##### Preparation of Eco-NaMnOx-Gg and Eco-NaMnOx-Ps

In a typical procedure, Eco-MnCl (30 g, 36 mmol of Mn, 1 eq.) was dissolved in distilled water (300 mL). H_2_O_2_ (40% *w*/*w* in H_2_O; 108 mmol, 3 eq.) was added while stirring. After 10 min, NaOH (19 M; 1.4 mmol, 40 eq.) was cautiously added. The suspension was stirred for 1.5 h at room temperature then filtered and washed three times with distilled water. The obtained solid was placed at 85 °C for 24 h, producing Eco-NaMnOx.

##### Preparation of Eco-MnOx-Ps Supported on MK10

Eco-MnOx-Ps (500 mg) and MK10 (1 g) were introduced into a flask containing distilled water (20 mL) at 90 °C while stirring. The mixture was stirred under reflux for 8 h, then filtered and washed three times with distilled water. The solid was placed at 85 °C for 24 h.

#### 3.2.2. Preparation of Citric Acid Functionalised Coffee Grounds

Coffee grounds were rinsed thoroughly with hot water until elimination of soluble products, then placed at 85 °C for 24 h. The resulting coffee grounds (5 g) and citric acid (4 g) were introduced into a flask containing anhydrous ethanol (20 mL) and stirred under reflux for 1 h. Ethanol was then evaporated, and the solid was placed at 120 °C for 12 h then the solid was put in distilled water (20 mL) at room temperature while stirring. After 15 min, the suspension was filtered and washed with distilled water. The resulting solid was placed at 85 °C for 24 h.

#### 3.2.3. General Procedures for the Synthesis of Oxyterpenes

#### Synthesis of ß-Pinene Oxide **2**

In a typical procedure, ß-pinene **1** (1.7 mmol, 1 eq.), ecocatalyst (8.5 μmol, 0.005 eq. of Mn) and NaHCO_3 (s)_ (8.5 mmol, 5 eq.) were introduced in a flask containing distilled water (20 mL) and acetone (20 mL) at 30 °C for 10 min. H_2_O_2 (aq.)_ (40% *w*/*w* in H_2_O; 8.5 mmol, 5 eq.) was added dropwise for 2 h. After 2 h, the suspension was filtered and extracted three times with ethyl acetate. The organic layers were collected together and dried using anhydrous MgSO_4_. The solvent was then evaporated. If the reaction was executed on a larger scale, ß-pinene oxide was isolated by distillation under vacuum with a cooled trap (see Supplementary Materials).

Isomer A (83%):

^1^H NMR (400 MHz, acetone_d6): δ (ppm): 0.93 (s 3H, H10); 1.24 (s, 3H, H9); 1.45 (t, 1H, H2, *J* = 5.33 Hz); 1.60 (d, 1H, H8a, *J* = 10.22 Hz); 1.65–1.74 (m, 1 H, H6a); 1.82–1.89 (m, 2H, H5); 1.94–2.00 (m, 1H, H4); 2.07–2.13 (m, 1H, H6b); 2.23–2.29 (m, 1H, H8b); 2.51 (d, 1H, H7a, *J* = 5.16 Hz); 2.68 (d, 1H, H7b, *J* = 5.16 Hz).

^13^C NMR (100 MHz, acetone_d6): δ (ppm): 21 (C10); 23 (C5); 24 (C6/C8); 26 (C6/C8); 26 (C9); 41 (C4); 42 (C3); 50 (C2); 56 (C7); 61 (C1).

IR: 1374, 1461, 2873, 2920, 2979, 3028 cm^−1^.

Isomer B (17%)

^1^H NMR (400 MHz, acetone_d6): δ (ppm): 0.93 (s 3H, H10); 1.24 (s, 3H, H9); 1.45 (t, 1H, H2, *J* = 5.33 Hz); 1.60 (d, 1H, H8a, *J* = 10.22 Hz); 1.65–1.74 (m, 1H, H6a); 1.82–1.89 (m, 2H, H5); 1.94–2.00 (m, 1H, H4); 2.07–2.13 (m, 1H, H6b); 2.23–2.29 (m, 1H, H8b); 2.38 (d, 1H, H7a, *J* = 5.16 Hz); 2.45 (d, 1H, H7b, *J* = 5.16 Hz).

^13^C NMR (100 MHz, acetone_d6): δ (ppm): 21 (C10); 22 (C5); 23 (C6/C8); 25 (C6/C8); 26 (C9); 41 (C4); 41 (C3); 49 (C2); 56 (C7); 61 (C1).

IR: 1374, 1461, 2873, 2920, 2979, 3028 cm^−1^.

#### Synthesis of Myrtenol **3**

In a typical procedure, ß-pinene oxide (**2**) (0.5 mmol) and a source of acid (2.5 mmol, 5 eq.) were introduced in a flask containing CPME (cyclopentyl methyl ether; 10 mL) under reflux. After 10 h, the suspension was filtered and washed three times with ethyl acetate. The filtrate was collected, dried using anhydrous MgSO_4_ and its solvent evaporated. Myrtenol was purified on a silica column using an eluant of 50% of ethyl acetate and 50% of petroleum ether.

^1^H NMR (500 MHz, acetone_*d*_6_): δ (ppm): 0.85 (s, 3H, H10); 1.17 (d, 1H, H8a, *J* = 8.52 Hz); 1.29 (s, 3H, H9); 2.05–2.15 (m, 2H, H2–H4); 2.18–2.34 (m, 2H, H5); 2.37–2.42 (m, 1H, H8b, *J* = 8.52 Hz, 5.57 Hz, 11.26 Hz); 3.91 (s, 2H, H7); 5.43 (m, 1H, H6).

^13^C NMR (500 MHz, acetone_*d*_6_): δ (ppm): 20.58 (C10); 25.80 (C9); 30.89 (C5); 31.33 (C8); 37.70 (C3); 41.05 (C4); 43.12 (C2); 64.60 (C7); 115.77 (C6); 148.74 (C1).

#### One pot Synthesis of 7-Hydroxy-α-terpineol 4 from β-Pinene **1**

Formation of 7-hydroxyterpineol **4** is a one-pot synthesis which follows directly ß-pinene epoxidation. In a typical procedure, ß-pinene **1** (1.7 mmol, 1 eq.), ecocatalyst (8.5 μmol, 0.005 eq. of Mn) and NaHCO_3 (s)_ (8.5 mmol, 5 eq.) were placed in a mixture of distilled water (20 mL) and acetone (20 mL) at 30 °C for 10 min while stirring. H_2_O_2 (aq.)_ (40% *w*/*w* in H_2_O; 8.5 mmol, 5 eq.) was added dropwise for 2 h using a syringe-pushing device. After 2 h, oxalic acid (13 mmol, 8 eq.) was added into the mixture. This was stirred for 1 more hour at 30 °C then filtered and extracted three times with ethyl acetate. The organic layers were collected together and extracted with a saturated solution of NaHCO_3_, then with brine before being dried using anhydrous MgSO_4_. The solvent was evaporated. 7-hydroxy-α-terpineol was purified on a silica column with a gradient of 100% cyclohexane to 100% ethyl acetate in 30 min followed by 10 min at 100% ethyl acetate.

^1^H NMR (400 MHz, acetone_*d*_6_): δ (ppm): 1.12 (s, 3H, H10); 1.14 (s, 3H, H9); 1.19–1,25 (m, 1H, H3a); 1.80–1.87 (m, 1H, H5a); 1.94 (m, 1H, H4, *J* = 17.11 Hz, 11.94 Hz, 5.16 Hz, 2.09 Hz); 1.96–2.00 (m, 2H, H2a–H3b); 2.08–2.14 (m, 2H, H2b–H5b); 3.13 (s, 1H, H12); 3.54 (t, 1H, H11); 3.86–3.93 (m, 2H, H7); 5.62 (s, 1H, H6).

^13^C NMR (100 MHz, acetone_d6): δ (ppm): 24 (C3); 27 (C10); 27 (C2/C5); 27 (C2/C5); 28 (C9); 46 (C4); 67 (C7); 72 (C8); 122 (C6); 139 (C1).

IR: 1674, 2842, 2864, 2885, 2923, 2976, 3285 cm^−1^.

#### Sequential Synthesis of 7-Hydroxy-α-terpineol 4 from β-Pinene Oxide **2**

In a typical procedure, ß-pinene oxide **2** (0.25 mmol, 1 eq.) was dissolved in distilled water (5 mL) and acetone (5 mL). Hydrochloric acid (0.11 mol.L^−1^, 3 eq.) was added and the reaction mixture was stirred at room temperature for 1 h. It was extracted three times with ethyl acetate. The organic layers were collected together and extracted with a saturated solution of NaHCO_3_, then with brine before being dried using anhydrous MgSO_4_. The solvent was evaporated.

#### Synthesis of Perillyl Alcohol **5**

In a typical procedure, ß-pinene oxide **2** (0.5 mmol) and catalyst (85 mg) were introduced in a flask containing CPME (20 mL) at room temperature. After 10 min, the suspension was filtered and washed three times with ethyl acetate. The filtrate was collected and its solvent was evaporated. Perillyl alcohol was purified on a silica column with a gradient of 100% cyclohexane to 90% cyclohexane: 10% ethyl acetate in 10 min followed by 10 min at 90% cyclohexane: 10% ethyl acetate.

^1^H NMR (400 MHz, acetone_*d*_6_): δ (ppm): 1.40–1.48 (m, 1H, H3a); 1.72 (s, 3H, H10); 1.79–1.84 (m, 1H, H3b); 1.87–1.96 (m, 1H, H5a); 2.07–2.17 (m, 4H, H2-H4-H5b); 3.66 (s, 1H, H_OH_); 3.86–3.93 (m, 2H, H7); 4.70 (s, 2H, H9); 5.64 (s, 1H, H6).

^13^C NMR (100 MHz, acetone_*d*_6_): δ (ppm): 21 (C10); 27 (C2); 28 (C3); 31 (C5); 42 (C4); 67 (C7); 109 (C9); 121 (C6); 139 (C1); 151 (C8).

IR: 1375, 1434, 1646, 2837, 2923, 2964, 3075, 3318 cm^−1^.

#### Synthesis of Perillaldehyde **6**

In a typical procedure, perillyl alcohol **5** (4 mmol, 1 eq.), Eco-NaMnOx-Ps (8 mmol, 2 eq. of Mn) and CuO (16 mmol, 4 eq.) were introduced in a flask containing cyclohexane (20 mL) under reflux and an O_2_ atmosphere for 2 h. The O_2_ atmosphere was ensured by using a bottle of dioxygene with an exit pressure of 0.1 mbar. The mixture was filtered and washed three times with ethyl acetate. The filtrate was collected, and its solvent was evaporated. Perillaldehyde was purified on a silica column using an eluant of 10% ethyl acetate and 90% of cyclohexane.

^1^H NMR (400 MHz, acetone_*d*_6_): δ (ppm): 1.40–1.47 (m, 1H, H3b); 1.76 (s, 3H, H10); 1.93–1.88 (m, 1H, H4); 2.11–2.14 (m, 1H, H3a); 2.29–2.23 (m, 2H, H2); 2.40–2.32 (m, 1H, H5b); 2.53–2.44 (m, 1H, H5a); 4.76–4.74 (m, 1H, H9b); 4.78–4.76 (m, 1H, H9a); 6.92 (m, 1H, H6); 9.44 (s, 1H, H7).

^13^C NMR (100 MHz, acetone_*d*_6_): δ (ppm): 20 (C10); 22 (C2); 27 (C3); 32 (C5); 41 (C4); 110 (C9); 142 (C1); 149 (C8); 151 (C6); 194 (C7).

IR: 888, 1166, 1376, 1435, 1643, 1680, 2720, 2811, 2929, 2966, 3079 cm^−1^.

#### Synthesis of Cuminaldehyde **7**

In a typical procedure, perillaldehyde **6** (0.5 mmol, 1 eq.), ecocatalyst (1.3 mmol, 2.6 eq. of Mn) and citric acid functionalised coffee grounds (500 mg) were introduced in a flask containing CPME (2 mL) under reflux. After 5 h, the suspension was filtered and washed three times with ethyl acetate. The filtrate was collected, and its solvent was evaporated. Cuminaldehyde was purified on a silica column using an eluant of 50% of diethyl ether and 50% of petroleum ether.

^1^H NMR (400 MHz, acetone_*d*_6_): δ (ppm): 1.26 (d, 6H, H9–H10, *J* = 6.87 Hz); 3.01 (spt, 1H, H8, *J* = 6.87 Hz); 7.48 (d, 2H, H3–H5, *J* = 8.35 Hz); 7.85 (d, 2H, H2–H6, *J* = 8.35 Hz); 9.99 (s, 1H, H7).

^13^C NMR (100 MHz, acetone_*d*_6_): δ (ppm): 23 (C9-C10); 128 (C3-C5); 131 (C2-C6); 136 (C1); 157 (C4); 192 (C7).

## 4. Conclusions

Although challenging because of their low stability, the synthesis of six cyclic oxyterpenes was achieved in moderate to good yields. The syntheses of ß-pinene oxide, perillyl alcohol and perillaldehyde led to yields equivalent to those found in the literature. In addition, new selective syntheses of myrtenol, 7-hydroxy-α-terpineol and cuminaldehyde are reported here for the first time.

The oxidation reactions were successfully catalysed using a new generation of ecocatalysts. This new generation of ecocatalysts, derived from Mn-rich water lettuce, presents a double environmental advantage, as the ecocatalysts are biosourced and the plant species is an invasive alien species. Its harvest is, therefore, valuable to the environment. For the reactions in which the ecocatalysts could not produce the desired product, other natural or biosourced catalysts were found to be efficient.

Moreover, only green solvents and renewable resources were systematically used in mild conditions for each of the six syntheses. Our strategy, based on using a toolbox of ecocatalysts, which combines synthetic performances and environmental benefits, integrates the pillars of sustainability.

## Figures and Tables

**Table 1:** Elemental composition of the ecocatalysts determined by MP-AES analyses.

		Composition (Weight% (±%RSD))						
Entry	Ecocatalyst	Mn	Ca	Fe	Mg	Na	K	Al
1	Roots of *P. stratiotes*	5.20 (4.97)	5.65 (4.60)	1.66 (1.64)	1.58 (0.83)	2.85 (2.06)	5.19 (2.60)	0.36 (0.36)
2	Eco-MnOx-Ps	7.53 (2.45)	10.56 (4.14)	2.37 (0.77)	3.54 (3.15)	4.57 (0.37)	9.64 (3.32)	0.56 (0.04)
3	Eco-MnCl-Ps	7.03 (2.07)	5.53 (2.67)	2.08 (1.02)	1.47 (0.74)	2.18 (0.88)	5.50 (1.74)	0.40 (0.20)
4	Eco-NaMnOx-Ps	15.94 (2.70)	13.75 (4.75)	5.27 (0.39)	3.59 (0.56)	1.24 (0.76)	0.04 (0.05)	0.89 (0.32)

**Table 2:** XRPD analyses of the ecocatalysts.

Entry	Ecocatalyst	Mn	Na	Ca	Si
1	Eco-MnOx-Ps	MnO_2_, K_2_Mn_4_O_8_	NaCl, KCl, K_3_Na(SO_4_)_2_	CaCO_3_	SiO_2_
2	Eco-MnCl-Ps	K_3_NaMnCl_6_	NaCl	-	-
3 [45]	Eco-CaMgOx-Gg	Ca_2_Mn_3_O_8_	K_3_Na(SO_4_)_2_, KCl	CaCO_3_	SiO_2_
4 [45]	Eco-MnCl-Gg	KMnCl_3_	NaCl	CaCl_2_	-

**Table 3:**
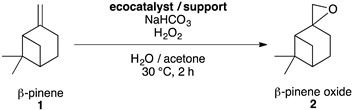
Synthesis of ß-pinene oxide **2** ^a^.

Entry	Ecocatalyst	Catalytic Loading (Eq.)	Support	Conv. (%) ^b^	Yield (%) ^b^
1	Eco-CaMnOx-Gg	0.005	/	89	33
2	Eco-MnOx-Ps	0.005	/	>99	63 ^c^
3		0.005	MK10	97	26
4		0.01	/	>99	43
5	Eco-MnCl-Ps	0.005	/	>99	63
6		0.005	MK10	79	35
7	/	/	MK10	0	0

^a^*ß*-pinene (1.7 mmol, 1 eq.), ecocatalyst (x eq.), NaHCO_3_ (8 mmol, 5 eq.), H_2_O_2_ (40 wt%, 8 mmol, 5 eq.) in H_2_O (20 mL): acetone (20 mL), 30 °C, 2 h. ^b^ Conversions and yields were determined by GCMS FID using biphenyl as an internal standard. ^c^ Isolated yield.

**Table 4:**
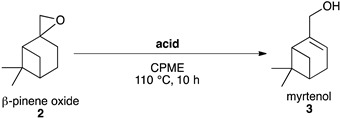
Selective opening of ß-pinene oxide **2** into myrtenol **3** ^a^.

Entry	Acid	Conv. (%) ^b^	Yield (%) *^b^*		
			3	4	5
1	betaine hydrochloride	>99	45 (44) ^c^	0	5
2	thiamine hydrochloride	>99	33	0	12
3	acetic acid	97	21	17	3
4	ascorbic acid	>99	10	0	20

^a^*ß*-pinene oxide (0.5 mmol, 1 eq.), acid (2.5 mmol, 5 eq.), CPME (10 mL), 110 °C, 10 h. ^b^ Conversions and yields were determined by GCMS FID using biphenyl as an internal standard. ^c^ Isolated yield.

**Table 5:**
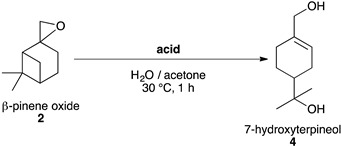
Selective opening of ß-pinene oxide **2** into 7-hydroxy-α-terpineol **4** ^a^.

Entry	Acid	Eq. of Acid	Conv. (%) ^b^	Yield (%) ^b^
1	HCl	1	66	31
2		3	>99	76
3	oxalic acid	3	>99	76(72) ^c^
4	formic acid	3	>99	73
5	citric acid	3	>99	65
6	betaine hydrochloride	3	>99	75
7	thiamine hydrochloride	3	>99	73

^a^*ß*-pinene oxide (1.7 mmol, 1 eq.), acid (x eq.), in H_2_O (20 mL): acetone (20 mL), 30 °C, 1 h. ^b^ Conversions and yields were determined by GCMS FID using biphenyl as an internal standard. ^c^ Yields after purification.

**Table 6:**
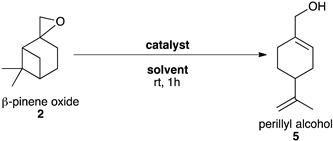
Selective opening of ß-pinene oxide **2** into perillyl alcohol **5** ^a^.

Entry	Catalyst	Catalytic Loading	Solvent	Conv. (%) ^b^	Yield (%) ^b^
1	Eco-MnCl-Ps	85 mg	CPME (2 mL)	>99	4
2	MK10	85 mg	DCM (2 mL)	>99	26
3			Me-THF (2 mL)	>99	34
4			CPME (2 mL)	>99	28
5	MK10	85 mg	Me-THF (50 mL)	>99	27
6			CPME (50 mL)	>99	40(38) ^c^
7	MK10	8.5 mg	CPME (50 mL)	>99	35
8		850 mg		>99	36

^a^ ß-pinene oxide (0.5 mmol), catalyst (x mg) in CPME (x mL), rt, 1 h. ^b^ Conversions and yields were determined by GCMS FID using biphenyl as an internal standard. ^c^ Yields after purification.

**Table 7:**
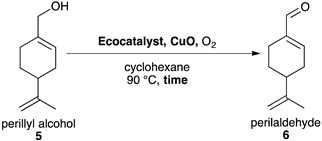
Synthesis of perillaldehyde **6** ^a^.

Entry	Ecocatalyst	Catalytic Loading (Eq.)	CuO (Eq.)	Time (h)	Conv. (%) ^b^	Yield (%) ^b^
1	Eco-NaMnOx-Ps	1	-	2	41	38
2		2	-		81	61
3		2	4		80	66(63) ^c^
4		4	8		94	45
5		-	4		0	0
6		2	4	4	84	61
7		2	4	7	78	49

^a^ perillyl alcohol (4 mmol, 1 eq.), Eco-NaMnOx-Ps (x eq.), CuO (x eq.) in cyclohexane (20 mL), O_2_ (0.1 mbar), 90 °C. ^b^ Conversions and yields were determined by GCMS FID using biphenyl as an internal standard. ^c^ Isolated yield.

**Table 8:**
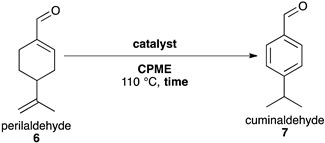
Synthesis of cuminaldehyde **7** ^a^.

Entry	Catalyst	CPME (mL)	Time (h) ^c^	Conv. (%) ^b^	Yield (%) ^b^
1	Eco-MnCl-Ps	0	2	>99	6
2	MK10			>99	5
3	Eco-MnCl-Ps + MK10			>99	5
4	Coffee ground-CA ^d^			0	-
5	Eco-MnCl-Ps + Coffee ground- CA ^d^			>99	9
6	Eco-MnCl-Ps	2	5	>99	27
7	Eco-MnCl-Ps + Coffee ground-CA ^d^			>99	29
8	Eco-MnCl-Ps	10	27	96	22
9	Eco-MnCl-Ps + Coffee ground-CA ^d^			>99	55(53) ^e^

^a^ Perillaldehyde (0.5 mmol), Eco-MnCl-Ps (1 g), Coffee ground-CA (1 g), CPME (x mL), 110 °C. ^b^ Conversions and yields were determined by GCMS FID using biphenyle as an internal standard. ^c^ Time to obtain a quantitative conversion. ^d^ coffee ground-CA stands for coffee ground that was functionalized with citric acid. ^e^ Yields after purification.

## Data Availability

Not applicable.

## Supplementary Materials

The following are available online. ^1^H NMR spectra and scale-up procedures.

## Sample Availability

Samples of the compounds are not available from the authors.

## References

[1] Gomes B.S., Neto B.P.S., Lopes E.M., Cunha F.V.M., Araújo A.R., Wanderley C.W.S., Wong D.V.T., Júnior R.C.P.L., Ribeiro R.A., Sousa D.P. (2017). Anti-Inflammatory Effect of the Monoterpene Myrtenol Is Dependent on the Direct Modulation of Neutrophil Migration and Oxidative Stress. Chem. Biol. Interact..

[2] Moreira M.R., Salvadori M.G., de Almeida A.A., de Sousa D.P., Jordán J., Satyal P., de Freitas R.M., de Almeida R.N. (2014). Anxiolytic-like Effects and Mechanism of (-)-Myrtenol: A Monoterpene Alcohol. Neurosci. Lett..

[3] de Britto R.M., da Silva-Neto J.A., Mesquita T.R.R., de Vasconcelos C.M.L., de Almeida G.K.M., de Jesus I.C.G., dos Santos P.H., Souza D.S., Miguel-dos-Santos R., de Sá L.A. (2018). Myrtenol Protects against Myocardial Ischemia-Reperfusion Injury through Antioxidant and Anti-Apoptotic Dependent Mechanisms. Food Chem. Toxicol..

[4] Belanger J.T. (1998). Perillyl Alcohol: Applications in Oncology. Altern. Med. Rev..

[5] Chen T.C., Fonseca C.O.D., Schönthal A.H. (2015). Preclinical Development and Clinical Use of Perillyl Alcohol for Chemoprevention and Cancer Therapy. Am. J. Cancer Res..

[6] Benedito R.B., Alves M.F., Pereira W.B., de Arruda Torres P., Costa J.P., da Rocha Tomé A., de Cássia da Silveira e Sá R., de Sousa D.P., Ferreira P.M.P., de Freitas R.M. (2017). Perillyl Alcohol: Antinociceptive Effects and Histopathological Analysis in Rodent Brains. Nat. Prod. Commun..

[7] Chastain D.E., Sanders W.E.S., Sanders C.C. (1992). Using Perillyl Alcohol to Kill Bacteria and Yeasts. U.S. Patent.

[8] Zafeer M.F., Firdaus F., Ahmad F., Ullah R., Anis E., Waseem M., Ali A., Hossain M.M. (2018). Perillyl Alcohol Alleviates Amyloid-β Peptides-Induced Mitochondrial Dysfunction and Cytotoxicity in SH-SY5Y Cells. Int. J. Biol. Macromol..

[9] Rodriguez A.A.M., Carvalho L.J.M., Kimura E.A., Katzin A.M. (2018). Perillyl Alcohol Exhibits in Vitro Inhibitory Activity against Plasmodium Falciparum and Protects against Experimental Cerebral Malaria. Int. J. Antimicrob. Agents.

[10] Surburg H., Panten J. (2016). Common Fragrance and Flavor Materials: Preparation, Properties and Uses.

[11] Bhatia S.P., McGinty D., Letizia C.S., Api A.M. (2008). Fragrance Material Review on P-Mentha-1,8-Dien-7-Ol. Food Chem. Toxicol..

[12] O’Brien-Nabors L. (2016). Alternative Sweeteners.

[13] Omari-Siaw E., Zhu Y., Wang H., Peng W., Firempong C.K., Wang Y.W., Cao X., Deng W., Yu J., Xu X. (2016). Hypolipidemic Potential of Perillaldehyde-Loaded Self-Nanoemulsifying Delivery System in High-Fat Diet Induced Hyperlipidemic Mice: Formulation, in Vitro and in Vivo Evaluation. Eur. J. Pharm. Sci..

[14] Xu L., Li Y., Fu Q., Ma S. (2014). Perillaldehyde Attenuates Cerebral Ischemia-Reperfusion Injury-Triggered Overexpression of Inflammatory Cytokines via Modulating Akt/JNK Pathway in the Rat Brain Cortex. Biochem. Biophys. Res. Commun..

[15] Ji W.-W., Wang S.-Y., Ma Z.-Q., Li R.-P., Li S.-S., Xue J.-S., Li W., Niu X.-X., Yan L., Zhang X. (2014). Effects of Perillaldehyde on Alternations in Serum Cytokines and Depressive-like Behavior in Mice after Lipopolysaccharide Administration. Pharmacol. Biochem. Behav..

[16] Tian J., Wang Y., Zeng H., Li Z., Zhang P., Tessema A., Peng X. (2015). Efficacy and Possible Mechanisms of Perillaldehyde in Control of Aspergillus Niger Causing Grape Decay. Int. J. Food Microbiol..

[17] Sato K., Krist S., Buchbauer G. (2006). Antimicrobial Effect of *Trans*-Cinnamaldehyde, (−)-Perillaldehyde, (−)-Citronellal, Citral, Eugenol and Carvacrol on Airborne Microbes Using an Airwasher. Biol. Pharm. Bull..

[18] Mandal M., Mandal S., Preedy V.R. (2016). Chapter 42—Cumin (*Cuminum cyminum* L.) Oils. Essential Oils in Food Preservation, Flavor and Safety.

[19] Hajlaoui H., Mighri H., Noumi E., Snoussi M., Trabelsi N., Ksouri R., Bakhrouf A. (2010). Chemical Composition and Biological Activities of Tunisian *Cuminum Cyminum* L. Essential Oil: A High Effectiveness against Vibrio Spp. Strains. Food Chem. Toxicol..

[20] Rihawy M.S., Bakraji E.H., Odeh A. (2014). PIXE and GC–MS Investigation for the Determination of the Chemical Composition of Syrian *Cuminum cyminum* L.. Appl. Radiat. Isot..

[21] Sahana K., Nagarajan S., Mohan Rao L.J., Preedy V.R., Watson R.R., Patel V.B. (2011). Chapter 50—Cumin (*Cuminum cyminum* L.) Seed Volatile Oil: Chemistry and Role in Health and Disease Prevention. Nuts and Seeds in Health and Disease Prevention.

[22] Wongkattiya N., Sanguansermsri P., Fraser I.H., Sanguansermsri D. (2019). Antibacterial Activity of Cuminaldehyde on Food-Borne Pathogens, the Bioactive Component of Essential Oil from *Cuminum Cyminum* L. Collected in Thailand. J. Complement. Integr. Med..

[23] Ebada M.E. (2017). Cuminaldehyde: A Potential Drug Candidate. J. Pharmcol. Clin. Res..

[24] Corma A., Renz M., Susarte M. (2009). Transformation of Biomass Products into Fine Chemicals Catalyzed by Solid Lewis- and Brønsted-Acids. Top. Catal..

[25] Vyskočilová E., Malý M., Aho A., Krupka J., Červený L. (2016). The Solvent Effect in β-Pinene Oxide Rearrangement. Reac. Kinet. Mech. Cat..

[26] Vyskočilová E., Dušek J., Babirádová M., Krupka J., Paterová I., Červený L. (2018). Perillyl Alcohol Preparation from β-Pinene Oxide Using Fe-Modified Zeolite Beta. Res. Chem. Intermed..

[27] Luštická I., Jansa P., Abelová L., Vyskočilová-Leitmannová E., Červený L. (2013). Immobilization of Pyridinium Nitrate and Its Application for the Catalytic Converting of β-Pinene Oxide. Reac. Kinet. Mech. Cat..

[28] Sánchez-Velandia J.E., Gelves J.-F., Dorkis L., Márquez M.-A., Villa A.-L. (2019). Ring-Opening of β-Pinene Epoxide into High-Added Value Products over Colombian Natural Zeolite. Microporous Mesoporous Mater..

[29] Sánchez-Velandia J.E., Mejía S.M., Villa A.L. (2020). Reaction Mechanism of the Isomerization of Monoterpene Epoxides with Fe^3+^ as Active Catalytic Specie: A Computational Approach. J. Phys. Chem. A.

[30] Escande V., Poullain C., Clavé G., Petit E., Masquelez N., Hesemann P., Grison C. (2017). Bio-Based and Environmental Input for Transfer Hydrogenation Using EcoNi(0) Catalyst in Isopropanol. Appl. Catal. B.

[31] Bihanic C., Richards K., Olszewski T.K., Grison C. (2019). Eco-Mn Ecocatalysts: Toolbox for Sustainable and Green Lewis Acid Catalysis and Oxidation Reactions. ChemCatChem.

[32] Bihanic C., Diliberto S., Pelissier F., Petit E., Boulanger C., Grison C. (2020). Eco-CaMnOx: A Greener Generation of Eco-Catalysts for Eco-Friendly Oxidation Processes. ACS Sustain. Chem. Eng..

[33] Clavé G., Garoux L., Boulanger C., Hesemann P., Grison C. (2016). Ecological Recycling of a Bio-Based Catalyst for Cu Click Reaction: A New Strategy for a Greener Sustainable Catalysis. ChemistrySelect.

[34] Clavé G., Pelissier F., Campidelli S., Grison C. (2017). Ecocatalyzed Suzuki Cross Coupling of Heteroaryl Compounds. Green Chem..

[35] Olszewski T.K., Adler P., Grison C. (2019). Bio-Based Catalysts from Biomass Issued after Decontamination of Effluents Rich in Copper—An Innovative Approach towards Greener Copper-Based Catalysis. Catalysts.

[36] Stanovych A., Balloy M., Olszewski T.K., Petit E., Grison C. (2019). Depollution of Mining Effluents: Innovative Mobilization of Plant Resources. Environ. Sci. Pollut. Res..

[37] Grison C., Adler P., Deyris P.-A., Diliberto S., Boulanger C. (2021). A Green Approach for the Reduction of Representative Aryl Functional Groups Using Palladium Ecocatalysts. Green Chem. Lett. Rev..

[38] Cases L., Adler P., Pelissier F., Diliberto S., Boulanger C., Grison C. (2021). New Biomaterials for Ni Biosorption Turned into Catalysts for Suzuki–Miyaura Cross Coupling of Aryl Iodides in Green Conditions. RSC Adv..

[39] Grison C., Lock Toy Ki Y. (2021). Ecocatalysis, a New Vision of Green and Sustainable Chemistry. Curr. Opin. Green Sustain. Chem..

[40] Deyris P.-A., Grison C. (2018). Nature, Ecology and Chemistry: An Unusual Combination for a New Green Catalysis, Ecocatalysis. Curr. Opin. Green Sustain. Chem..

[41] Grison C. (2015). Special Issue in Environmental Science and Pollution Research: Combining Phytoextraction and EcoCatalysis: An Environmental, Ecological, Ethic and Economic Opportunity. Environ. Sci. Pollut. Res..

[42] Grison C., Escande V. (2020). Use of Certain Manganese-Accumulating Plants for Carrying out Organic Chemistry Reactions. U.S. Patent.

[43] Grison C., Carrasco D., Stanovych A. (2018). Procede De Preparation De Materiau D’origine Vegetale Riche En Acides Phenoliques, Comprenant Au Moins Un Metal, Pour La Mise En Oeuvre De Reactions De Synthese Organique.

[44] Grison C. (2015). Procédé de Préparation D’acides Mineraux D’origine Naturelle et Utilisation des Acides Obtenus en Synthèse Organique.

[45] Garel C., Fonda E., Michalowicz A., Diliberto S., Boulanger C., Petit E., Legrand Y.M., Poullain C., Grison C. (2019). Structure and Composition of First Biosourced Mn-Rich Catalysts with a Unique Vegetal Footprint. Mater. Today Sustain..

[46] Neuenschwander U., Meier E., Hermans I. (2011). Peculiarities of β-Pinene Autoxidation. ChemSusChem.

[47] Coelho J.V., Oliveira L.C.A., Moura F.C.C., de Souza P.P., Silva C.A., Batista K.B., Silva M.J. (2012). da β-Pinene Oxidation by Hydrogen Peroxide Catalyzed by Modified Niobium-MCM. Appl. Catal. A-Gen..

[48] da Silva M.J., Vieira L.M.M., Oliveira A.A., Ribeiro M.C. (2013). Novel Effect of Palladium Catalysts on Chemoselective Oxidation of β-Pinene by Hydrogen Peroxide. Monatsh. Chem..

[49] Schröder K., Junge K., Spannenberg A., Beller M. (2010). Design of a Bio-Inspired Imidazole-Based Iron Catalyst for Epoxidation of Olefins: Mechanistic Insights. Catal. Today.

[50] Constantino M.G., Júnior V.L., Invernize P.R., Filho L.C., José da Silva G.V. (2007). Opening of Epoxide Rings Catalyzed by Niobium Pentachloride. Synth. Commun..

[51] Fomenko V.V., Bakhvalov O.V., Kollegov V.F., Salakhutdinov N.F. (2017). Catalytic Epoxidation of β-Pinene with Aqueous Hydrogen Peroxide. Russ. J. Gen. Chem..

[52] Zheng W., Tan R., Zhao L., Chen Y., Xiong C., Yin D. (2014). Mn^2+^/Graphene Oxide Nanocomposite Efficiently Catalyzes the Epoxidation of Alkenes with H_2_O_2_. RSC Adv..

[53] McKillop A., Kabalka G.W., Reddy M.S. (2008). Sodium Perborate. Encyclopedia of Reagents for Organic Synthesis.

[54] Zheng W., Hu H., Chen Y., Tan R., Yin D. (2019). Diamine-Decorated Graphene Oxide with Immobilized Gold Nanoparticles of Small Size for Alkenes Epoxidation with H_2_O_2_. Catal. Lett..

[55] de Oliveira A.A., da Silva M.L., da Silva M.J. (2009). Palladium-Catalysed Oxidation of Bicycle Monoterpenes by Hydrogen Peroxide in Acetonitrile Solutions: A Metal Reoxidant-Free and Environmentally Benign Oxidative Process. Catal. Lett..

[56] Mouret A., Leclercq L., Mühlbauer A., Nardello-Rataj V. (2013). Eco-Friendly Solvents and Amphiphilic Catalytic Polyoxometalate Nanoparticles: A Winning Combination for Olefin Epoxidation. Green Chem..

[57] Martinez Q H., Amaya Á.A., Paez-Mozo E.A., Martinez O F., Valange S. (2021). Photo-Assisted O-Atom Transfer to Monoterpenes with Molecular Oxygen and a DioxoMo(VI) Complex Immobilized on TiO_2_ Nanotubes. Catal. Today.

[58] Li Q., Kung L., Peng S., Li X., Wang L. (2007). Synthesis of Perillaldehyde via Selective Oxidation of Perilla Alcohol. J. Nat. Sci.-Hunan Norm. Univ..

[59] Escande V., Renard B.-L., Grison C. (2015). Lewis Acid Catalysis and Green Oxidations: Sequential Tandem Oxidation Processes Induced by Mn-Hyperaccumulating Plants. Environ. Sci. Pollut. Res..

