# Supplementary material for: New Sustainable Synthetic Routes to Cyclic Oxyterpenes Using the Ecocatalyst Toolbox
Source: Molecules. 2021 Nov 27;26(23):7194. doi: 10.3390/molecules26237194 (PMC8658900; doi:10.3390/molecules26237194)
Supplement: Supplementary file 1 [file molecules-26-07194-s001.zip › molecules-1465077-supplementary.pdf]

New sustainable synthetic routes to cyclic oxyterpenes using the Ecocatalyst toolbox - Supporting informations.

Camille Bihanic [1†], Arthur Lasbleiz [1†], Morgan Regnier [1], Eddy Petit [2], Pierre Le Blainvaux [3], and Claude Grison [1,3,*]

1 Laboratory of Bio-inspirated Chemistry and Ecological Innovations (ChimEco), UMR 5021, CNRS – University of Montpellier
Cap Delta, 1682 rue de la Valsière, 34790 Grabels, France
2 European Institute of Membrane (IEM), UMR 5635 – University of Montpellier
163 rue Auguste Broussonet, 34090 Montpellier, France
3 BioInspir
Cap Delta, 1682 rue de la Valsière, 34790 Grabels, France

* Correspondence: claude.grison@cnrs.fr
† These authors contributed equally to this work.

Table des matières

I. **^{1}H NMR spectra……………..……………………………………………...……..….2**

II. **Scale-up procedures……………………………………………………….…………3**

I. ¹H NMR Spectra

β-pinene oxide in acetone-d₆

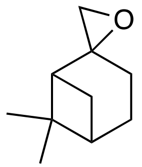

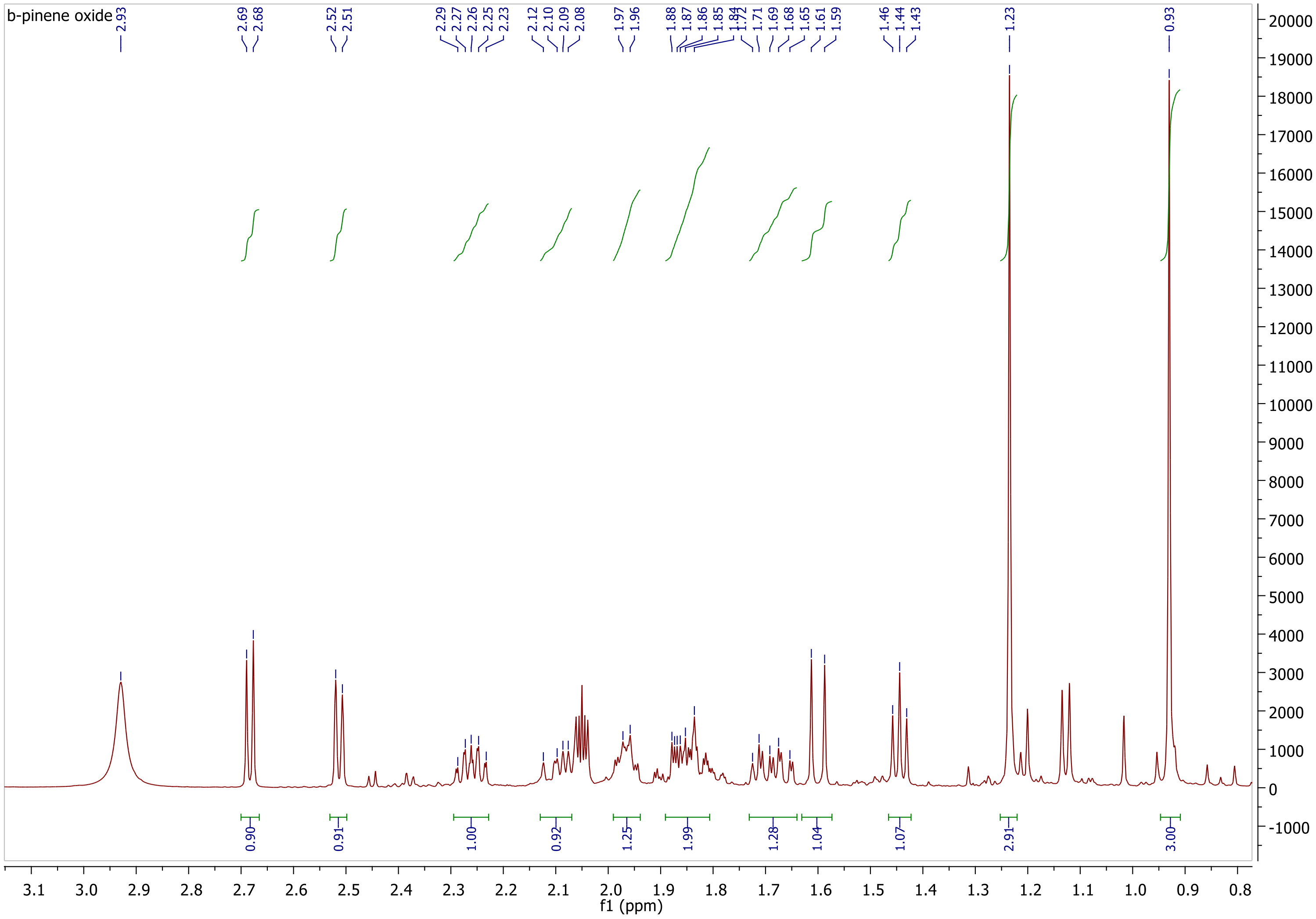

Myrtenol in acetone-d_6

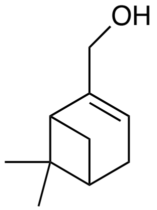

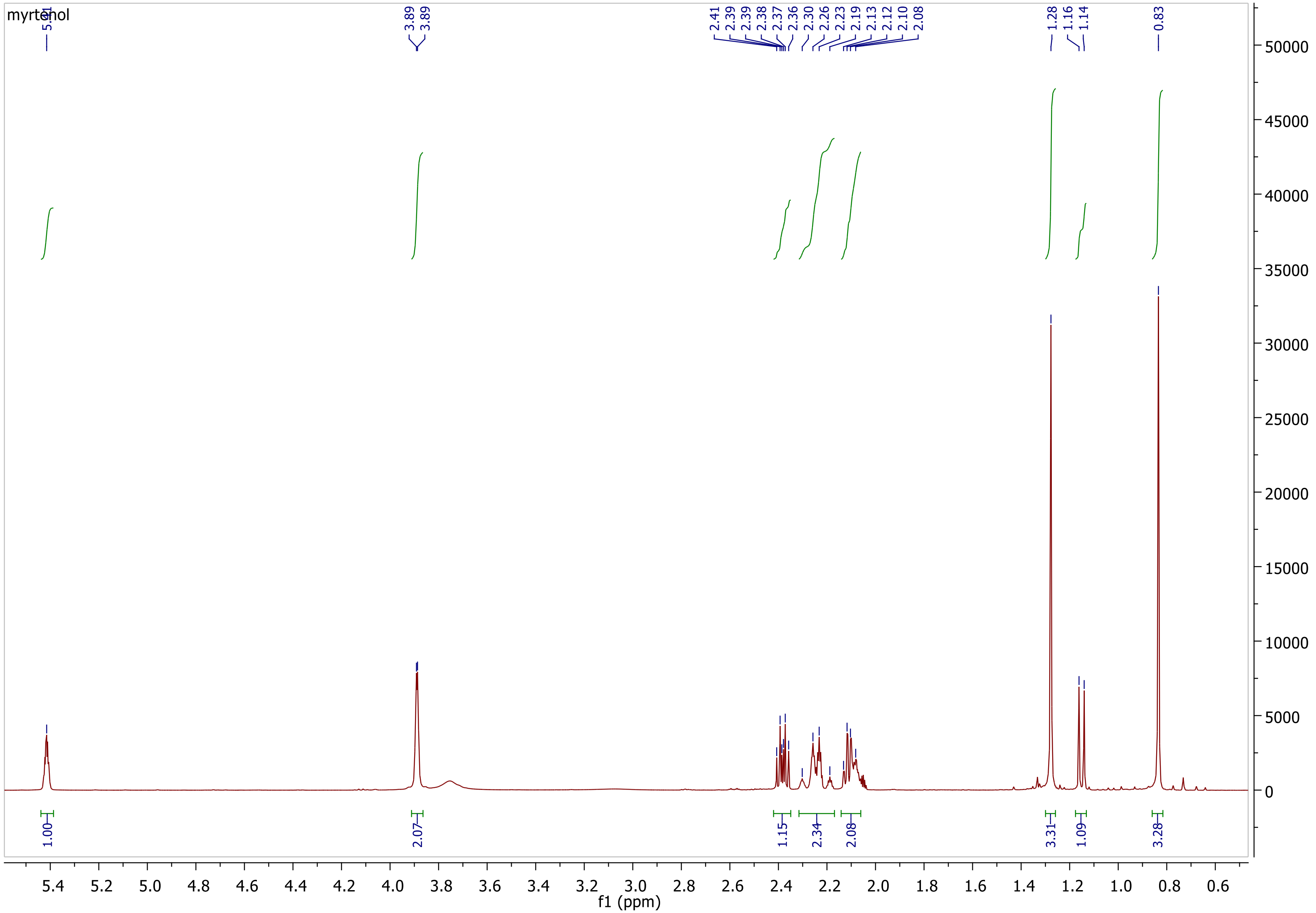

7-Hydroxyterpineol in acetone-d6

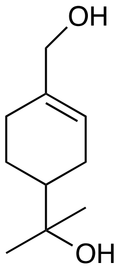

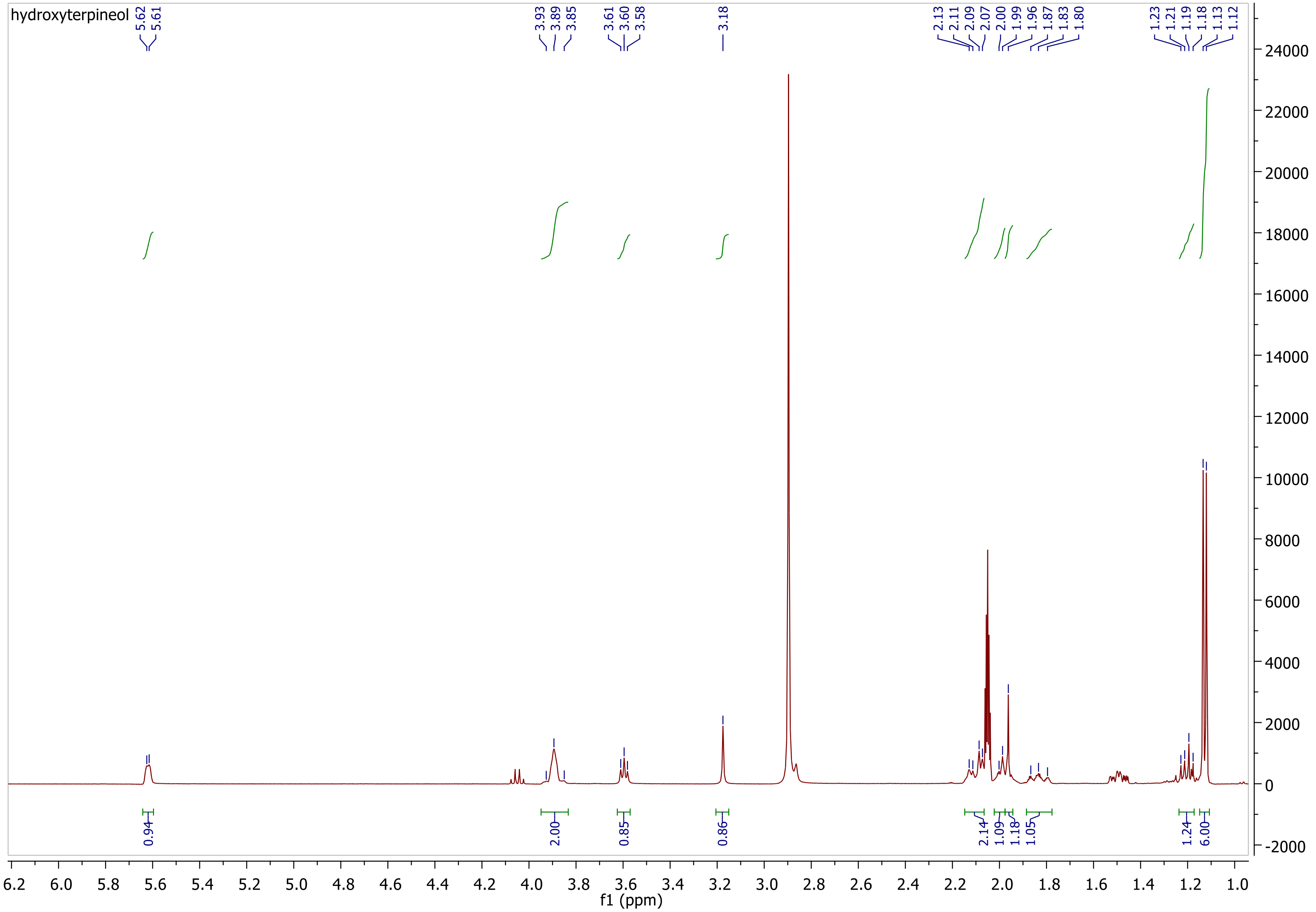

Perillyl alcohol in acetone-d_6

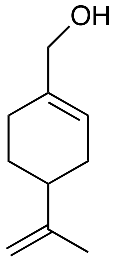

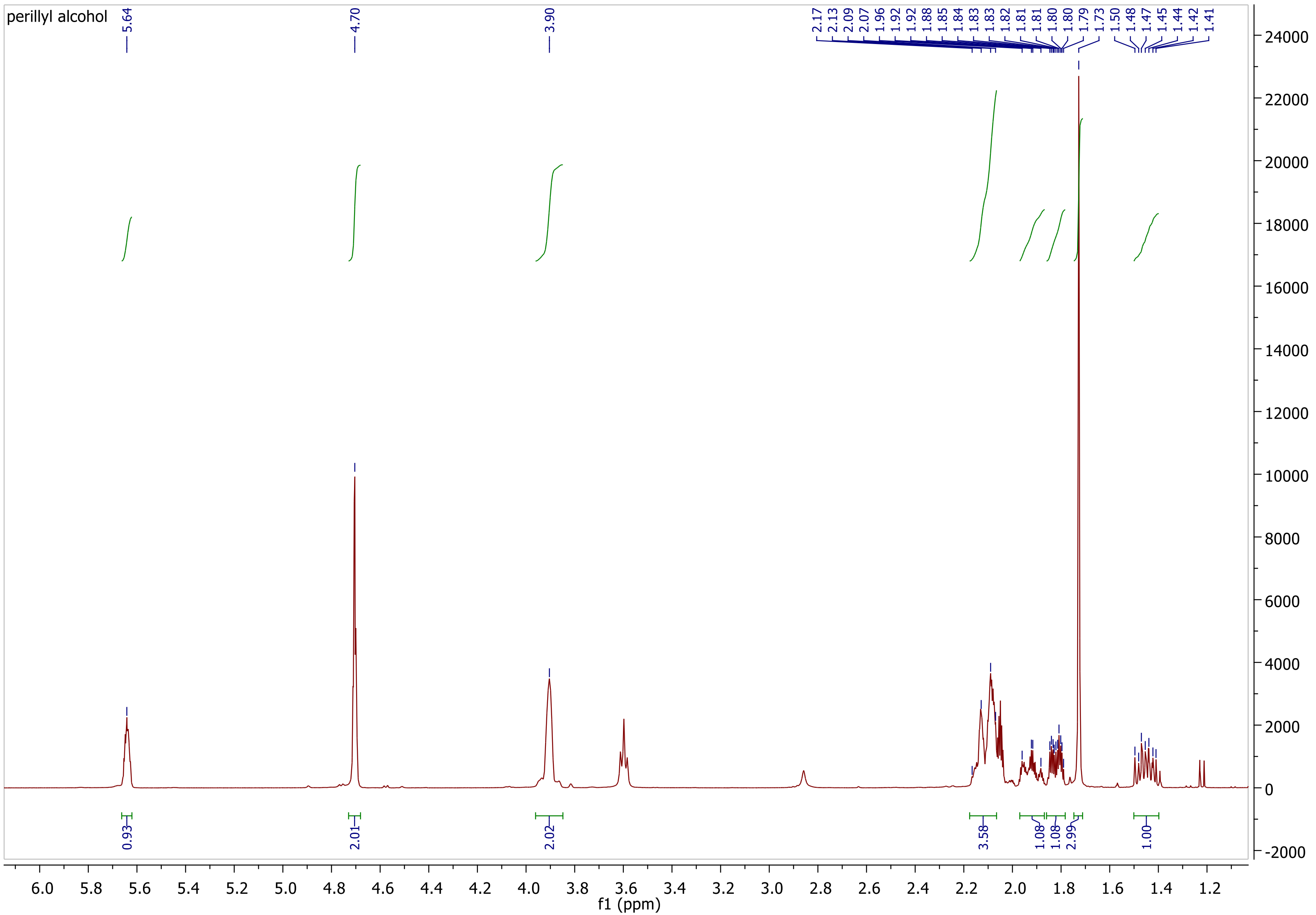

Perillaldehyde in acetone-d_6

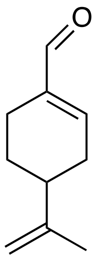

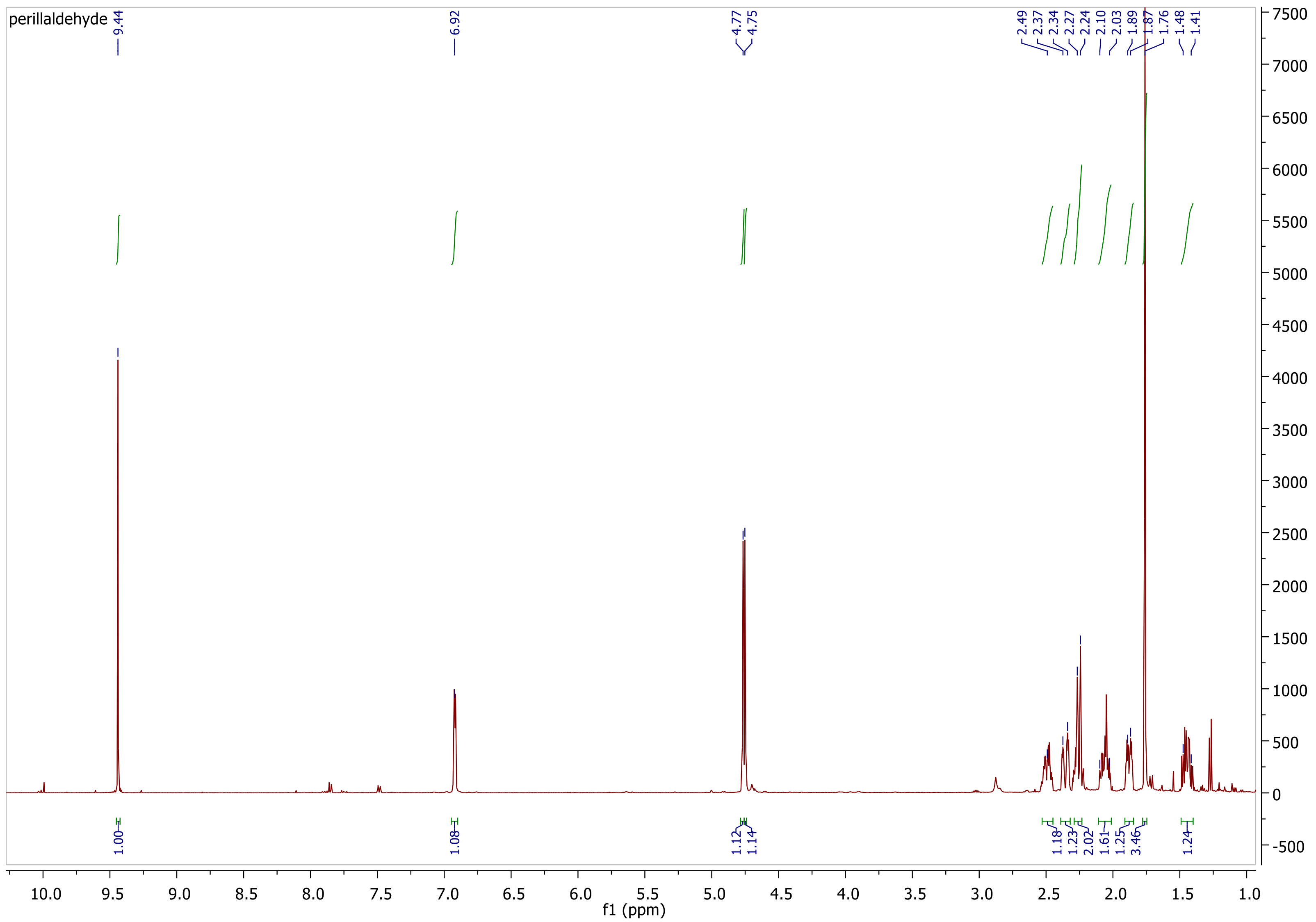

Cuminaldehyde in acetone-d_6

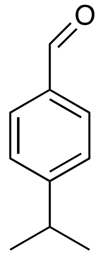

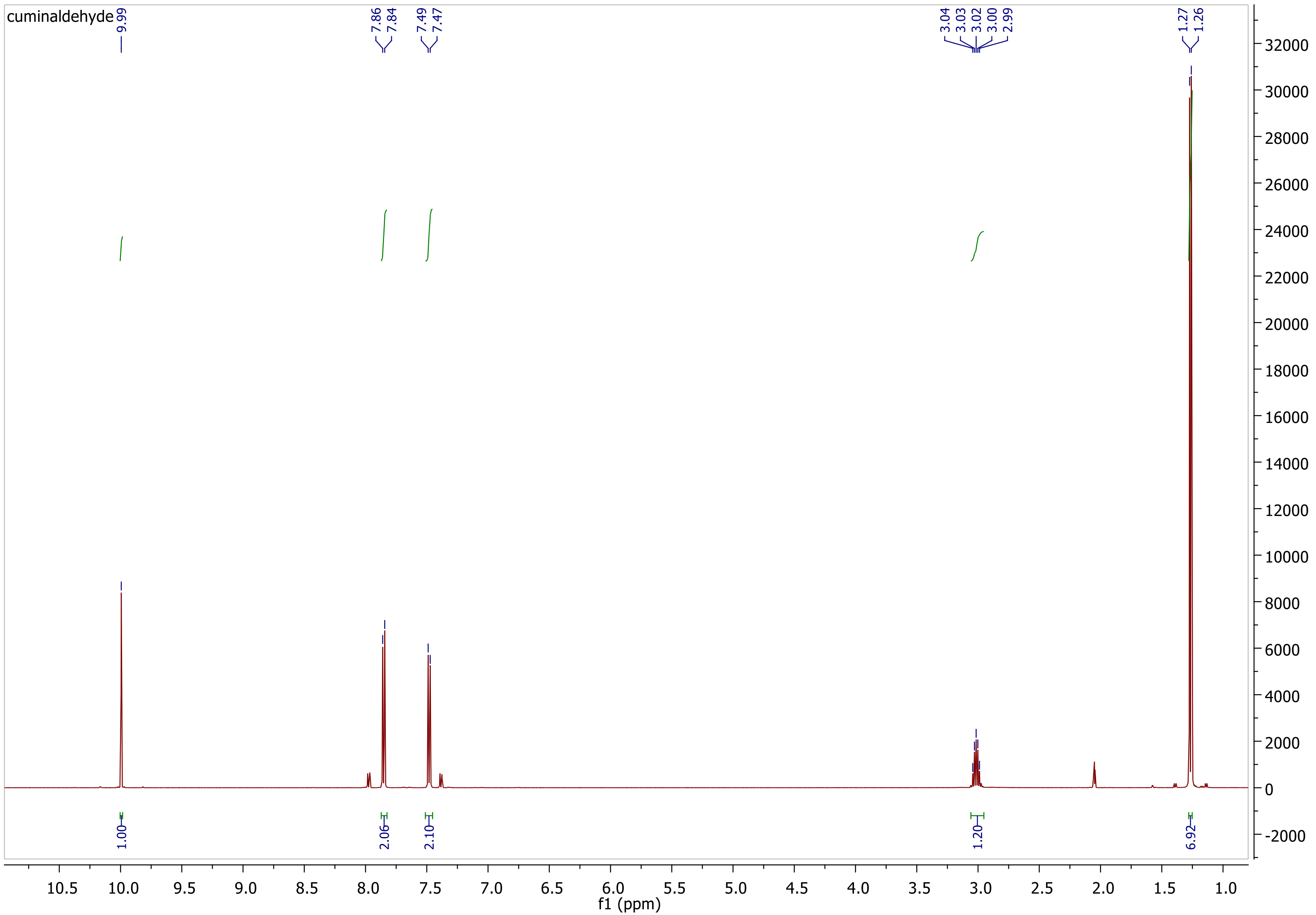

II. Scale up procedures

Scale-up for the synthesis of ß-pinene oxide **2**.

ß-pinene **1** (38 mmol, 1 eq.), ecocatalyst (200 µmol, 0.005 eq. of Mn) and $NaHCO_{3\ (aq)}$ (200 mmol, 5 eq.) were introduced in a flask containing distilled water (120 mL) and acetone (120 mL) at 30 °C for 10 minutes. $H_2O_{2\ (aq)}$ (40 % w/w in H_2O; 200 mmol, 5 eq.) was added dropwise for 2 h. After 2 h, the suspension was filtered and extracted three times with ethyl acetate. The organic layers were collected together and dried using anhydrous $MgSO_4$. The solvent was then evaporated. The crude was distilled under vacuum with a cooled trap.

Scale-up for the one pot synthesis of 7-hydroxyterpineol **4** from ß-pinene **1**.

For the one-pot synthesis, ß-pinene **1** (38 mmol, 1 eq.), ecocatalyst (200 µmol, 0.005 eq. of Mn) and $NaHCO_{3\ (s)}$ (200 mmol, 5 eq.) were placed in a mixture of distilled water (120 mL) and acetone (120 mL) at 30 °C for 10 minutes while stirring. $H_2O_{2\ (aq.)}$ (40 % w/w in H_2O; 200 mmol, 5 eq.) was added dropwise for 2 h using a syringe-pushing device. After 2 h, oxalic acid (304 mmol, 8 eq.) was added into the mixture. It was stirred for 1 more hour at 30 °C. It was then filtered and extracted three times with ethyl acetate. The organic layers were collected together and extracted with a saturated solution of $NaHCO_3$, then with brine before being dried using anhydrous $MgSO_4$. The solvent was evaporated.
